# Summary of third annual MCBK public meeting: Mobilizing computable biomedical knowledge—Accelerating the second knowledge revolution

**DOI:** 10.1002/lrh2.10255

**Published:** 2020-12-23

**Authors:** Michelle Williams, Rachel L. Richesson, Bruce E. Bray, Robert A. Greenes, Leslie D. McIntosh, Blackford Middleton, Gerald Perry, Jodyn Platt, Christopher Shaffer

**Affiliations:** ^1^ Department of Learning Health Sciences University of Michigan Medical School Ann Arbor Michigan USA; ^2^ University of Utah School of Medicine Salt Lake City Utah USA; ^3^ College of Health Solutions, Arizona State University Phoenix Arizona USA; ^4^ Research Data Alliance, London, England and Ripeta Saint Louis Missouri USA; ^5^ Apervita Chicago Illinois USA; ^6^ University of Arizona Libraries, University of Arizona Tucson Arizona USA; ^7^ UCSF Library University of California San Francisco California USA

**Keywords:** computable biomedical knowledge, dissemination, metadata, standards, clinical decision support, HIT policy

## Abstract

The volume of biomedical knowledge is growing exponentially and much of this knowledge is represented in computer executable formats, such as models, algorithms, and programmatic code. There is a growing need to apply this knowledge to improve health in Learning Health Systems, health delivery organizations, and other settings. However, most organizations do not yet have the infrastructure required to consume and apply computable knowledge, and national policies and standards adoption are not sufficient to ensure that it is discoverable and used safely and fairly, nor is there widespread experience in the process of knowledge implementation as clinical decision support. The Mobilizing Computable Biomedical Knowledge (MCBK) community was formed in 2016 to address these needs. This report summarizes the main outputs of the third annual MCBK public meeting, which was held virtually from June 30 to July 1, 2020 and brought together over 200 participants from various domains to frame and address important dimensions for mobilizing CBK.

## 1 BACKGROUND

Despite the growing pace of biomedical knowledge,^1^ the United States continues to see increasing health disparities and decreasing life expectancies for some populations.^2^ The number of potential treatments and evidence base is steadily increasing but it is difficult to disseminate and implement these into practice, due to a number of challenges including volume, relevance to particular patients, and need to adapt to particular workflows and EHR technologies used where health‐related decisions are made. To be widely disseminated and actionable, knowledge needs to be distributed in usable and implementable formats—ie, computable biomedical knowledge (CBK). CBK, such as predictive models, rules, alerts, clinical pathways, or data visualizations, is necessary for the interventional approach of a learning health system. The mobilization of CBK can result in rapid mass access to computable knowledge with the potential to improve the health of individuals and populations on a large scale.^3^ We believe that a new, coordinated ecosystem is needed to revolutionize how knowledge and evidence can be distributed to support decision‐making and action and thereby benefit human health.

While work has proceeded in this space for many years, the movement to mobilize computable biomedical knowledge (MCBK) was conceived 3 years ago by a number of thought leaders. The MCBK movement aims to achieve better health in diverse settings by widely sharing knowledge in a computable format.^4^, ^5^ In this report, we present a summary of the third annual MCBK public meeting held virtually from June 30 to July 1, 2020.

## 2 MEETING AND PARTICIPANT INFORMATION

Due to the COVID‐19 pandemic, the meeting was held as an online interactive conference, replacing the live meeting originally planned to take place at the National Institutes of Health in Bethesda, Maryland. Close to 200 people were registered before the event and 239 unique participants [RR1] joined virtually, representing the following types of organizations:

Universities/Academic Medical Centers (n = 93 [39%]).

Commercial/Industry and consultants (n = 65 [27%]).

Multidisciplinary clinicians (n = 25 [10%]).

Government (n = 16 [7%]).

Other (n = 17[7%]).

Professional societies (n = 9 [6%]).

Health plans or providers (n = 6 [4%]).

Students/Fellows (n = 3[1%].

## 3 MEETING STRUCTURE AND OVERVIEW

A multidisciplinary Steering Committee^6^ guided the selection of topics and activities. The virtual meeting included remarks from national leaders, panel presentation, and a lightning round of poster sessions and technical demonstrations. There were also breakout sessions for work groups and poster presentations. The meeting agenda, list of speakers, registered participants, and presentations are available at www.mobilizecbk.org. Slides and videos for all talks are available at https://mobilizecbk.med.umich.edu/events/2020-mcbk-virtual-meeting.

An in‐person meeting was not possible due to the COVID‐19 pandemic and restrictions on travel and interpersonal contact. A professional AV service was used to set up an IBM platform for streaming the meeting live to viewers. This technology enabled individual speakers and groups of presenters to share with the audience. Audience and attendees could submit questions to a central technology manager who would relay them to speakers for answering. The Zoom platform, which enabled audience participation, was used for breakout meetings with the different work groups and for the poster session and social hour aspects of the meeting.

## 4 MEETING SPEAKERS AND CONTENT

### 4.1 Welcome remarks and keynote

Dr Rachel Richesson and Dr Charles (Chuck) Friedman, MCBK Steering Committee co‐chairs, opened the meeting with brief remarks on the fundamental principles of the MCBK community and its connection to the second Knowledge Revolution. They emphasized the MCBK commitment to address health problems across a wide range of domains through mass action, and addressed the role of libraries in enabling the dissemination of computable and actionable knowledge at a rapid pace. Drs Richesson and Friedman presented the meeting goals: to strengthen the foundation of shared recognition and principles for mobilizing CBK, to advance Work Group action plans, to generate new ideas and identify opportunities for future collaboration and to grow the MCBK community.

Dr Patricia Brennan, Director of the National Library of Medicine (NLM), gave an opening keynote address emphasizing the importance the NLM places on CBK, especially in the midst of the COVID‐19 pandemic. Dr Brennan described the redesign of PubMed to present knowledge (references) in order most relevant to users, rather than chronological. This can facilitate getting users the right knowledge for their needs amid the growing volume of knowledge. She stressed the NLM's commitment to advanced analytics and said the NLM's initiatives to improve implementation guidelines for clinical data research and care intersects with the work of MCBK. Dr Brennan emphasized NLM's commitment to learning more about the impact of accelerated discovery and the open sharing of research results on scholarly communications. Also, their many activities/projects that support the data, tools, methods, and policies around AI and ML to ready our nation for that change. Finally, she shared plans of the NLM to support data access and knowledge creation activities through a number of Data Design Centers, which would ensure data quality and access, transparency about bias, and development of tools to enable/empower more consumers to generate knowledge to address persistent and emergent health problems.

### 4.2 Panel presentations

The 2‐day meeting included five different panels to address different perspectives and stakeholder groups for CBK. These included library, research, demonstrations (existing platforms and communities for CBK), international perspectives, and patient perspectives.

#### 4.2.1 CBK and library of the future panel: Anticipating a second knowledge revolution

Because of the obvious and foundational role of libraries in the distribution of knowledge, a panel of librarians and information experts described different aspects of the library's role in the MCBK. The four‐person panel provided descriptive examples of how libraries can lead the future mobilization of CBK. The panel was co‐moderated by Christopher Shaffer and Terrie Wheeler.

Dr Kristi Holmes[RR2] presented an approach of building on infrastructure, inclusiveness, and operationalized processes by bringing together data, informatics tools, people, and perspectives. Sharing her work building with CTSA support, other multidisciplinary research programs, and community engagement activities, her premise is that libraries empower collaborative ecosystems.

Ms Violeta Ilik discussed technical systems in the Knowledge Revolution, stressing that libraries should be constantly readapting in order to provide enriching experiences of sharing knowledge via research, critical inquiry, teaching, learning, publishing, or innovation.

Dr Jeff Oliver introduced the concept of building capacity for computational literacy. He described the importance of human capital and digital science literacies including the capability of using programs such as R and Python to make data computable for use. He shared his work training faculty and staff on “the carpentries”—incorporating training for building skills for data literacy and software development into health center and general academic library offerings.

Mr Bart Ragon modeled library science as the key to moving knowledge forward. Mr Ragon emphasized the growing value of open‐source contribution and presented an example of merging data sources to determine publication impact. In his talk, he charged our audience to examine what the roles of the CBK‐enabled “library of the future” should be: gatekeeper, creator, or consumer of CBK?

#### 4.2.2 CBK revolutionizing biomedical research

A panel with two presenters moderated by Dr Friedman presented on how computable formats can be integrated and shared by health information systems and applications. Dr Grace Peng described how her work intersects with the MCBK vision. She presented applied computable knowledge treatment models using examples from medical comorbidities associated with lower back pain. Dr Peng also outlined the promotion of medical simulation research capable of evaluating skills acquisition, outcome assessment, and technology development.

Dr Herbert Sauro described the way biomedical models are currently published (or not!), emphasizing that the future should be long‐term model repositories and technologies to manage published models—with adequate information and metadata to be reproducible. He likened the current human readable journal article to an “advertisement” for a study or model description but noted future implementers and health information consumers need more resources to evaluate or apply these models. Dr Sauro called PubMed a host that could lead the move to the digital preservation of models.

#### 4.2.3 Computable Knowledge infrastructure in action

The four‐person panel moderated by Dr Peter Embi discussed what it takes to mobilize knowledge artifacts and the main obstacles that are necessary to overcome in order to build a digital ecosystem.

Dr Linn Brandt presented the MAGIC* Evidence Ecosystem Foundation (*MAGIC = MAking GRADE the Irresistible Choice) as a case study on how structured guidelines feed into the biomedical knowledge community to aid decision support. Dr Brandt stressed the importance of creating trustworthy guidelines in the digital ecosystem that works to create guidance, disseminate data, implement evidence, and improve practice.

Dr Saverio Maviglia described the requirements for managing knowledge infrastructure as: depth, provenance and metadata, versioning and lifecycle, dependency management, search and query, validate and test and learning.

Dr Allen Flynn spoke about standardizing CBK to drive an open infrastructure of the future. Dr Flynn said he is motivated by a future decentralized web approach for infrastructure, which would allow wide access and use of complex information through a digital object Knowledge Grid.^6^ He described the dual view of CBK artifacts as resources and services.

Dr Blackford Middleton addressed the knowledge management, dissemination, and execution approach taken at Apervita, building upon standards from HL7. Apervita creates a commercial platform and marketplace that facilitates knowledge sharing and delivery. Dr Middleton explained that there is value to co‐locate data and knowledge in the cloud so that knowledge services can be inserted into a variety of endpoints.

#### 4.2.4 The state of the MCBK in US and related global movements

As part of the Annual Meeting goal to grow collaboration and build relationships with entities of similar interests, three speakers described their organization's synergies with the MCBK movement.

Dr Friedman addressed the multi‐stakeholder nature of the MCBK movement, emphasizing its core value of including a wide range of stakeholders across the domains of health and biomedicine. He laid out plans to take MCBK global and explained the movement's future considerations, which include a future organizational home, bringing in all stakeholders, and defining long‐term value propositions.

Dr Phillip Scott and Dr Jeremy Wyatt described the 2019 inaugural meeting of MCBK United Kingdom (UK). The co‐chairs of MCBK UK said the main conclusion from the first MCBK UK meeting was the significance of CBK to future health systems, which should be supported and developed with cross‐sector support from informatics and clinical experts.

Steve Bernstein addressed the FAIR (Findable, Accessible, Interoperable, Reusable) principles^7^ and objectives of the Agency for Healthcare Research and Quality (AHRQ) evidence‐based Care Transformation Support (ACTS) initiative. Mr Bernstein explained the ACTS stakeholder‐driven approach and described the AHRQ digital knowledge platform as the point of intersection with the MCBK community.

#### 4.2.5 Stakeholder engagement

The two‐person panel moderated by Joshua Rubin presented the importance of diversity and accessibility to movements like MCBK.

Michael Fitts outlined how the experience he had when he was first diagnosed with Parkinson's disease led him to advocate for building better partnerships between patients and providers. He emphasized the benefit of having CBK that is derived from representative research and would be relevant to diverse populations.

Sally Okun described ways to ensure the democratization of CBK, referring to six person‐informed principles that will be a foundation for CBK implementation. The principles she focused on were continuous and shared learning; respect and empowered individual choice; informed and understood consent; people first governance; open communication and accountability; and inclusivity, diversity, and equity.

### 4.3 Poster session

Twenty‐six posters were presented via an online lightning round on Day 1 of the meeting. There were 10 technical posters grouped into active CBK themes of standards, platforms, methods, or applications. Another 15 project posters were divided into two themes: Foundations and Applied, presented in a lightning round. Poster authors also expanded on their posters at the designated poster session. At the end of Day 1, presenters were able to expand on the posters at a virtual breakout session.

Of the 26 posters, 5 (19%) came from commercial entities, 17 (65%) from academia, 2 (8%) from government, and 2 (8%) from standards development organizations. Collectively, the posters represented the perspectives of knowledge developers, disseminators, and users. Poster abstracts from the meeting are included in this issue and digital posters can be viewed here: https://mobilizecbk.med.umich.edu/events/2020-mcbk-virtual-meeting.

## 5 WORKGROUP ACTION SESSIONS AND ACTIVITY

The speakers described above‐provided background, vision, and motivation for meeting participants, who were charged to advance the MCBK vision through the four work groups formed during the first MCBK public meeting. One breakout session (3 hours on Day 2) was designated as a Work Group Action Session. The work groups and their co‐chairs, scope, and discussions are summarized below.

The Standards Work Group SWG), led by Drs. Robert Greenes and Bruce Bray, is focused on identifying existing and emerging standards that will facilitate widespread use of knowledge by enabling FAIR^8^ and Trust capabilities.^7^ During the breakouts at the meeting, the SWG discussed efforts to define a set of nonoverlapping categories of metadata that will enable the FAIR+T capabilities. Prior to the SWG session, a team had been involved (ongoing since then) in defining these categories and using exemplar artifacts of different types to help elucidate them. It should be noted that the categories, which included Type, Biomedical Domain (now called Domain), Purpose, and Coverage (now called Evidential Basis), each have multiple dimensions of metadata, so a task is that of clarifying what those dimensions are, which is best done by examining artifacts. Also note that 7 other categories, for a total of 11 were briefly presented. After an introduction to the challenge, participants were asked to join separate sub‐breakouts, each examining the four categories, and to report back at the end of their breakouts. The Standards Work Group members, under the leadership of Dr Allen Flynn, are developing a publication that describes the metadata categories (currently totaling 13) needed to characterize CBKs. Participants in the session were encouraged to join the SWG's activities on an ongoing basis. Promoting continued collaboration among the various informatics standards and knowledge implementation groups, including coordination among the MCBK working groups, was emphasized as an important continuing goal.

The Technical Infrastructure Work Group, led by Dr Leslie McIntosh and Mr Chris Shaffer, and now transferred to Dr Jamie McCusker, is producing a white paper that will identify technical requirements for organizations to use and evaluate CBK. They are collecting real‐world examples and mini‐use cases to incorporate into their white paper. Components of the paper were discussed in the breakout session, including identifying the framework components necessary to move CBK from generation into practice by facilitating the testing, versioning, use, evaluation, scalability, interoperability, and dissemination of MCBK. Other questions discussed included: how do we build and share a conceptual infrastructure model that supports MCBK? What does the developer community need from the MCBK TI working group?

The Policy and Coordination to Ensure Quality and Trust Work Group discussions were led by co‐chairs Jodyn Platt and Blackford Middleton. This Work Group builds upon the significant conceptual and consensus work of the AHRQ‐funded Patient‐centered CDS Learning Network: Trust Framework Working Group in the area of establishing trustworthy knowledge artifacts used in clinical decision support.^9^ The group is focused on identifying and addressing gaps in policy and issues that would impact the quality or trustworthiness of CBK. During the breakouts, the group discussed the current and evolving landscape of emerging knowledge commons, and considered implications for governance and trust, as well as market considerations, business models, and governance strategies for organizations mobilizing CBK in real‐world settings. These issues were reviewed in small and large group discussions of a survey that is part of the research plan for the working group.

The Sustainability for Mobilization and Inclusion Work Group, chaired by Christine Dymek (now transferred to Terrie Wheeler) and Jerry Perry, continued discussion on curation of a list of important MCBK stakeholder groups, and the need to develop strategies and approaches to message and engage with each. The breakout included discussions of various approaches to work in partnership with professional societies and associations with a shared interest in CBK, considering roles both formal such as an appointed liaison to more informal, functional activities such as engaging as an advocate or “ambassador.” The Work Group considered models of engagement including memoranda of understanding and looked at examples in use for societies in the field of librarianship. The Work Group was reminded that although these groups might see the positive societal impact of MCBK, MCBK is abstract at this point and the business case is not always clear. This Work Group, therefore, will continue to develop value propositions that align with societies' business incentives, as well as jargon‐free and relatable messages that focus on how MCBK can alleviate current “pain points.” The Work Group pivoted then to consider advocacy around MCBK through scholarship and discussed topics of potential interest around which to develop a literature review. A subgroup was formed to actively pursue a review of the literature on the very broad topic of the “democratization of information” noting the Group's ongoing interest in MCBK and inclusion and equity.

## 6 CLOSING AND REFLECTIONS

The meeting closed with reflections from invited members of the MCBK Steering Committee, Dr Doug Van Houweling. Dr Van Houweling shared his thoughts on the wide range of CBK perspectives such as libraries, research, infrastructure, and stakeholders in addition to summarizing the breadth of the poster topics. He highlighted the robustness of the MCBK movement and said it represents a challenge on how best to respond to the opportunities, challenges, and responsibilities necessary to harness the collective expertise of the community moving forward. Referring back to the MCBK manifesto's statement on the potential of knowledge to improve health care, the health of individuals, and the health of populations, Dr Van Houweling underlined the community's challenge to imagine the audacity of its proposal and identify the gaps that need to be closed so as to meet the audaciousness of the MCBK manifesto. The gaps he identified were support from incumbents, cohesion, funding, infrastructure, technology, and recognition. Dr Van Houweling described the importance of the convening power of the MCBK community, evidenced by the large meeting attendance. He suggested the MCBK community should define its role in the movement and posed the questions of whether it should be convening the community, promoting CBK, building CBK infrastructure, or facilitating the movement. He stated that any one of the roles would be useful, but whatever that choice, it is important to identify the partners needed to engage and what would provide the most impact.

## 7 NEXT STEPS

MCBK continues to fill an important but broad niche based on the diversity of meeting attendees. Work group chairs and members plan to continue their activities into the next year and support plans for subsequent public meetings.

The University of Michigan will continue to provide communications support for MCBK workgroups and their members. A webinar was presented in Fall 2020 to summarize the meeting and workgroup action sessions. Plans for a Fourth Annual MCBK public meeting for Summer 2021 are underway. The MCBK is an open and inclusive community. Anyone that is interested in joining an MCBK Work Group may sign up here: http://mobilizecbk.org/.

## CONFLICT OF INTEREST

Blackford Middleton is employed by Apervita, Inc., which provides a platform and marketplace to democratize healthcare data and analytics. Leslie McIntosh is founder and CEO of Ripeta, LLC, a company providing reproducibility checks on scientific manuscripts. Gerald (Jerry) Perry served on the Board of Directors for the Association of Academic Health Sciences Libraries (AAHSL) during the period of work described in this article. AAHSL is one of the four professional societies of whose members were interviewed to develop targeted communications strategies for affinity professional societies, as a component of the Sustainability for Mobilization and Inclusion Work Group's efforts. Mr Perry has no competing commercial interests. The other authors (Rachel Richesson, Bruce Bray, Christine Dymek, Robert Greenes, Leslie McIntosh, Jodyn Platt, Christopher Shaffer, and Michelle Williams) have no competing interests to declare.
